# mSphere of Influence: Adenosine in Host Defense against Bacterial Pneumonia—Friend or Foe?

**DOI:** 10.1128/mSphere.00326-19

**Published:** 2019-07-10

**Authors:** Elsa N. Bou Ghanem

**Affiliations:** a Department of Microbiology and Immunology, University at Buffalo School of Medicine, Buffalo, New York, USA

**Keywords:** CD73, *Streptococcus pneumoniae*, bacterial pneumonia, extracellular adenosine, inflammation, lung infection, neutrophils

## Abstract

Elsa N. Bou Ghanem works in the field of innate immune senescence, inflammation, and host defense. In this mSphere of Influence article, she reflects on how “Adenosine A_2B_ receptor deficiency promotes host defenses against Gram-negative bacterial pneumonia” by Barletta et al. (K. E. Barletta, R. E. Cagnina, M. D. Burdick, J. Linden, and B. Mehrad, Am J Respir Crit Care Med 186:1044–1050, 2012, https://doi.org/10.1164/rccm.201204-0622OC) impacted her own work examining the role of the extracellular adenosine pathway in neutrophil responses and host defense against pneumococcal pneumonia.

## COMMENTARY

It is a truth universally acknowledged that successful host defense balances inflammatory responses to clear invading pathogens with a return to homeostasis to avoid tissue injury (1, 2). Extracellular adenosine is a well-known regulator of inflammation that is produced in the extracellular environment as a breakdown product of ATP released from injured cells by the sequential action of two extracellular enzymes, CD39 and CD73 (3). Extracellular adenosine can signal via four G-protein-coupled receptors, A1, A2A, A2B, and A3, which are ubiquitously expressed on host cells (3). Although very well studied in tissue injury models (3), the role of adenosine in host defense against pathogens was not well explored until fairly recently (4). In a 2012 study titled “Adenosine A_2B_ receptor deficiency promotes host defenses against Gram-negative bacterial pneumonia,” Barletta and colleagues explored the role of A2B receptor in host defense against Klebsiella pneumoniae lung infection (5). They found that signaling via the A2B receptor impaired host resistance by impairing neutrophil antibacterial activity.

To address the role of A2B in host defense, the authors used a mouse model of K. pneumoniae lung infection. They found that lack of A2B increased host survival following infection, which was attributed to a better ability to control pulmonary bacterial numbers and limit systemic spread of the infection. In exploring mechanisms, they found that A2B expression on hematopoietic cells was detrimental to the host. As A2B is known to be expressed on neutrophils and to regulate their influx into the airspace, the authors examined pulmonary neutrophil influx following infection but surprisingly found no significant difference in the recruitment of these cells to the lungs. Interestingly, A2B^−/−^ neutrophils were much better at killing K. pneumoniae than wild-type neutrophils due to their increased ability to form neutrophil extracellular traps (NETs). These findings were in contrast to acute lung injury models where A2B protected the host by modulating neutrophil recruitment (6–10), highlighting that pathogens change the dynamics of the immune response and that the role of the adenosine pathway may differ in sterile injury from that in the context of infection.

This paper came out at the beginning of my postdoctoral training and really shaped the questions and projects I would go on to pursue. At the time, I was working on the age-driven decline in host defense against Streptococcus pneumoniae (pneumococcus) (11), which remains the leading cause of community-acquired pneumonia in the elderly (12). I was focused on the role of neutrophils and what controls their recruitment and function during pneumococcal pneumonia and how that changes with aging. When this paper came out exploring a pathway that controlled neutrophil responses during bacterial pneumonia, it grabbed my attention. I remember thinking “what is extracellular adenosine?” After delving into the literature, I found that this was a well-established regulator of inflammation that had been thoroughly explored in hypoxia-induced lung injury among several other tissue injury models (13, 14) and is thought to mediate its effect by targeting leukocyte recruitment (15). Importantly, there were pharmacological inhibitors and agonists of the different enzymes and receptors with some of them in consideration for clinical use, which made this pathway very attractive to study (16). Surprisingly, the role of this pathway in lung infections was undercharacterized, so I tested the effect of pharmacologically blocking CD73, the enzyme required for extracellular adenosine production, on host defense against S. pneumoniae using a murine lung infection model. I was thrilled when I found that inhibiting CD73 resulted in more than a thousandfold increase in bacterial burdens in the lungs of mice. And so, a new project was born. We went on to find that extracellular adenosine production and signaling were crucial for host defense against pneumococcal pneumonia and that they were required for both resolution of pulmonary neutrophil influx and the antipneumococcal function of these immune cells (17). On the surface, adenosine seems to play opposing roles in pneumococcal pneumonia versus K. pneumoniae lung infection (5). However, extracellular adenosine is recognized by four different G-protein-coupled receptors that can inhibit or increase production of cAMP, are known to have opposing functions leading to enhanced or diminished acute inflammation, and can boost or blunt neutrophil antimicrobial responses (18). As adenosine receptors have various affinities to their ligand, work in my lab is exploring the hypothesis that these receptors have a time-dependent role in infection where at the onset of S. pneumoniae infection and resulting tissue damage, a moderate rise in extracellular adenosine results in selective engagement of the high-affinity receptors A1 and/or A3 mediating host protection, while continued damage and adenosine buildup later on stimulate the lower-affinity A2A and/or A2B receptors.

Around the time that the K. pneumoniae paper came out and following that, several papers exploring the role of the extracellular adenosine pathway in pulmonary infections were published. These include studies elucidating the role of CD73 and A1 in influenza A virus infection (19, 20), the role of CD73 during Mycobacterium tuberculosis infection (21), the role of CD73 in S. pneumoniae infections (17, 22), and the role of CD39 in Pseudomonas aeruginosa infection (23). In most of the above studies, extracellular adenosine modulated pathogen clearance and/or lung damage by controlling the recruitment of leukocytes and/or regulating their antimicrobial functions. The effect of adenosine during infection seems to be organ and adenosine receptor specific, either boosting or impairing host defense (4). In conclusion, it is now better appreciated that the extracellular adenosine pathway plays a major role in shaping the outcome of infections, and for our group, work by Barletta et al. paved the way for exploring the role of this pathway during bacterial pneumonia.
